# Bioprospecting for Thermozymes and Characterization of a Novel Lipolytic Thermozyme Belonging to the SGNH/GDSL Family of Hydrolases

**DOI:** 10.3390/ijms23105733

**Published:** 2022-05-20

**Authors:** Juan-José Escuder-Rodríguez, María-Eugenia DeCastro, Almudena Saavedra-Bouza, María-Isabel González-Siso, Manuel Becerra

**Affiliations:** EXPRELA Group, Advanced Scientific Research Center (CICA), Department of Biology, Faculty of Sciences, Universidade da Coruña, 15071 A Coruña, Spain; j.escuder@udc.es (J.-J.E.-R.); m.decastro@udc.es (M.-E.D.); almudena.saavedra@udc.es (A.S.-B.)

**Keywords:** bioprospection, metagenomes, thermozymes, SGNH/GDSL, hydrolases

## Abstract

Functional screenings were conducted on two metagenomic libraries from hot springs in order to find novel thermozymes with potential biotechnological applications. These included enzymes acting on plant cell walls such as endoglucanases and exoglucanases, β-glucosidases, xylanases, and β-xylosidases, and broad application enzymes such as proteases and lipolytic hydrolases. Of all the enzymes found by this bioprospection, we selected a novel lipolytic enzyme for further characterization. The protein was found to belong to the SGNH/GDSL family of hydrolases. It was purified and its biochemical parameters determined. We found that the enzyme was most active at 60 °C and pH 9 using pNP-laurate as substrate and was highly thermostable. It also showed preference for short-chained substrates and activation with temperature and with certain detergents such as Tween 80. Proteins of this family of hydrolases are relevant for their broad substrate specificity, that coupled with this protein’s high temperature optima, broad pH range, and thermostability further highlights its biotechnological potential.

## 1. Introduction

The function-driven approach to studying metagenomes is a powerful tool for the bioprospection of extreme environments. It consists of the cloning of metagenomic DNA in a suitable host to construct a library, that is later used to perform screenings in which the specific desired activity is assayed. This strategy has been extensively reviewed [1,2,3,4,5,6,7,8,9], often comparing it to the sequence-based approach where the metagenomic DNA is sequenced with Next-Generation Sequencing techniques. Due to the need for high computational power and its processing costs, as well as Whole Genome Sequencing costs and requirement for specific equipment, functional metagenomics can be regarded as a more affordable (yet still expensive and labor-demanding) method for assessing the metagenome. Moreover, the functional approach has some other advantages, such as the lack of requirement for previous knowledge on the gene product amino acid sequence; thus holding the potential to discover truly novel enzymes harboring new protein domains or belonging to not yet described protein families.

Many authors have identified main bottlenecks that functional screening studies usually face [1,10,11]. First, the isolation of contaminant-free environmental DNA of sufficient quality and integrity, in large-enough amounts and with minimal sampling bias is a challenging task, especially when the source material typically yields low biomass as is the case for thermal springs and other extreme habitats [3]. Particular to the function-driven strategy, the selection of a suitable heterologous host and vector system and of the screening method are key choices to successfully find sought activities [1,11]. The most obvious (but not to be overlooked) decision is to pick a host strain lacking the desired activity that would be screened (either naturally or by genetically engineering knock-out strains), as background signal can prevent discrimination between positive and negative hits [5]. It is generally accepted that the use of broad range shuttle vectors is desirable for the construction of metagenomic libraries as it may circumvent problems related to the heterologous host system by completely changing the cellular framework in which the gene products are being expressed [7,11,12,13,14]. The most widely used host for metagenomic libraries is *E. coli* [6,15], which has been tested for its expression potential against a set of different genomes, revealing a wide range that goes as low as 7% to as high as 73% of expressed genes [16].

Regarding the screening method, direct phenotypic detection is the most widespread method for functional screening of metagenomic libraries [11], using chromogenic or fluorescent substrates to detect enzymatic activities of interest, or substrates that leave a clear halo around the colonies producing the desired gene product [7]. The development of novel enzymatic assays has been driven by the need for more sensitivity and targeting a broader range of biotechnologically interesting activities [10,17].

Since their discovery, thermophilic microorganisms that thrive in high temperature environments such as hot springs have been considered an important source for thermozymes, as they have evolved and adapted their metabolism in harsh environmental conditions [18]. Even more, thermozymes with higher thermal stability and optimal temperatures than the optimal growth temperature of source thermophilic microorganisms are commonplace [19]. Many industrial applications can benefit from the use of thermozymes. Advantages such as lower operational costs and increased selectivity with a reduction in undesired by-products can be achieved by the use of thermally stable catalysts [18], replacing chemical processes or mesophilic variants. The mechanisms underlying the improved thermal stability and optimal activity rates at higher temperatures have been studied [19,20,21], and other resistances and tolerances towards other extreme conditions are often associated with thermozymes, such as activity at extreme pH values, and resistance to chemical denaturing agents and non-aqueous solvents [22] as well as proteolytic resistance [18,20] which optimally will align with the requirements of the industrial processes. Operation at high temperatures has other associated benefits, such as lowering the risk of contamination from microorganisms (most pathogenic and food-rotting microorganisms cannot survive temperatures above 70 °C) [20], lowering the viscosity of fluids allowing to reduce costs of processes such as pumping and filtration and increasing the heat and mass transfer rates as well as solubility of substrates (allowing for equilibriums shifts towards reaction products) [19,20].

Metagenomic surveys have successfully retrieved several thermozymes from high temperature habitats including hot springs [10], and comprehensive lists are available showing the continuous interest in these bioprospecting studies for the past two decades [3,6,9,15,23,24]. Many of the thermozymes sought are plant cell wall degradation enzymes, acting on cellulose, the most abundant polymer on Earth, and other complex polymers such as hemicellulose. These include cellulases, β-glucanases, xylanases, and β-xylanases as well as other accessory enzymes such as certain esterases [6,25,26,27]; but there is interest in many other glycosidases [18,19] such as β-galactosidases [28]; amylases and chitinases [9]; pullulanases [20]; pectinases [29]; as well as other non-carbohydrate related enzymes such as proteases [24], phosphatases and oxidorreductases [6], among others. Potential applications for these thermozymes are very varied. Cellulases (endoglucanases, exoglucanases, and β-glucanases) and hemicellulases (xylanases and β-xylosidases) are important in the process of biofuel (bioethanol) production from lignocellulosic biomass [6,24,30,31] and some cellulases are used in the formulation of detergents, among many other applications including paper and pulp, textile, and food processing industries [32,33]; lipases can be used in biodiesel production, production of cosmetics, processing in leather and pulp industries, synthesis of drugs and fine chemicals, and bioremediation [6,24,30,31,34] while some esterases are accessory enzymes in the lignocellulosic biomass degradation process [25], and proteases are components for detergents and can be used for processing in food, leather, pharmaceutical, and textile industries [6,24,30,31].

In the present work, we centered our efforts on screening two metagenomic libraries for a range of different biotechnologically relevant activities that mainly relate to plant biomass valorization [25,26,29] using the functional metagenomics approach. These include cellulases and β-glucosidades, xylanases, β-xylosidases, and feruloyl esterases. We also included more broad substrate hydrolases, namely lipolytic enzymes and proteases. As the sources for the metagenomic DNA for both libraries were hot springs, it was expected that the targeted gene products would have high temperature optima and thermostability, among potentially other beneficial characteristics from an industrial applicability standpoint. After detecting positive hits for some of these activities, we proceeded to undertake the heterologous expression, purification, and biochemical characterization of one of the lipolytic enzymes found.

## 2. Results

### 2.1. Water Sampling

Water from the As Burgas fountains had a measured temperature of 67 °C and pH 7.5. For Muiño da Veiga, the temperature was 68 °C and pH was 7.0. Previous reports [35,36] from the same sites reported very similar yet slightly lower temperatures (66.3 °C for As Burgas and 66 °C for Muiño da Veiga), similar or identical pH values for As Burgas (7.56 and 7.5), and varying pH values for Muiño (7.6 and 8.1). Thus, both hot springs can be described as thermophilic and circumneutral to slightly alkaline.

### 2.2. Library Construction

After cloning, the final library size was 27,898 clones for the As Burgas source, with clones distributed in 16 different 96-well plates, averaging 20 clones per well. For the Muiño da Veiga source, we obtained 4399 individual colonies, that were distributed in 5 different 96-well plates, averaging 10 clones per well. Other libraries using the similar commercial fosmid vectors pCC1FOS or pCC2FOS have been reported to yield similar numberw of clones [6], from as low as 5000 with the pCC2FOS vector (similar to our Muiño da Veiga library) to as big as 96,000 with the pCC1FOS fosmid, averaging 37,575 clones.

### 2.3. Functional Screening

**Figure 1:**
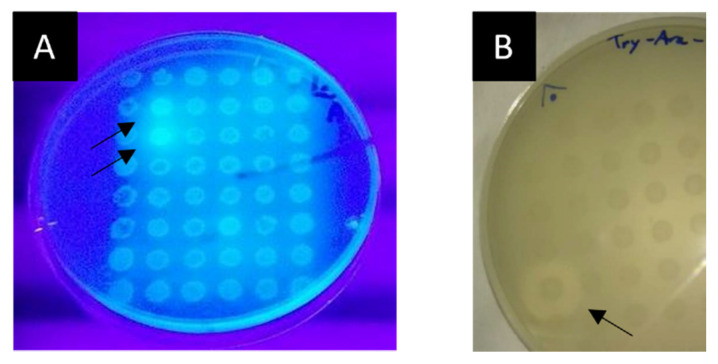
Activity screening results. (**A**) β-Xylosidase activity screening with two positive hits from the As Burgas Metagenomic library using MUX as the substrate. Observation under UV light for fluorescence around the colonies is required to identify positive hits. The black arrows indicate the location of two positive hits. (**B**) Lipolytic activity screening with a positive hit from the As Burgas metagenomic library using glyceryl tributyrate as the substrate. Direct observation of halos surrounding the colonies possessing the activity allows to identify positive hits. The black arrow points towards the positive hit.

An overview of all the functional screening results is given in Table 1. Examples of positive hit identification in the functional screenings are provided in Figure 1.

#### 2.3.1. Screening for Plant Cell Wall Degrading Enzymes

We found no positive exoglucanase clones using the screening protocols with AVICEL as the substrate in the two libraries. We also found no xylanase activity-conferring genes by screening the two metagenomic libraries using plates containing the AZCL-Xylan substrate.

No positive hits were found using the methods to find endoglucanases with CMC or AZCL-HE-Cellulose as substrates (solid media plate chromogenic substrate assays), but 2 As Burgas clones and 4 Muiño da Veiga clones were found positive for endoglucanase activity using the EnzChek fluorescent substrate (liquid media fluorogenic assays). Differences in the substrates’ detection thresholds are expected between chromogenic and fluorogenic molecules, especially when low amounts of enzymes are produced. The format of the screening changing from colonies on agar plates to 384-well plates can also improve the sensitivity of the method [8,13] and explain our different results between both methods.

Screening for β-xylosidase activity using the fluorogenic substrate MUX revealed 2 positive hits from the As Burgas metagenomic library but did not reveal any clone with that activity in the Muiño da Veiga library. For β-glucosidase activity using pNP-glucopyranoside as substrate, we found 5 positive hits for the As Burgas library and only 1 for the Muiño da Veiga library.

#### 2.3.2. Screening for Proteases

In total, 4 positive clones were found using Bodipy Casein fluorescent substrate in the As Burgas metagenomic library and 6 in the library from Muiño da Veiga.

#### 2.3.3. Screening for Lipolytic and Feruloyl Esterase Activities

The screening in the As Burgas metagenomic library using the glyceryl tributyrate substrate gave a single positive hit (labelled LipB12_A11). Additionally, we found 8 different clones harboring lipolytic activities in the Muiño da Veiga library. Subsequent streak cultures on screening media allowed the isolation of single colonies harboring the lipolytic activity. The following screening for feruloyl esterase activity of each of these clones using the fluorescent substrate MUTMAC did not yield any positive hits.

### 2.4. Subcloning of Selected Positive Lypolitic Activity-Conferring Genes and Sequence Analysis

**Figure 2:**
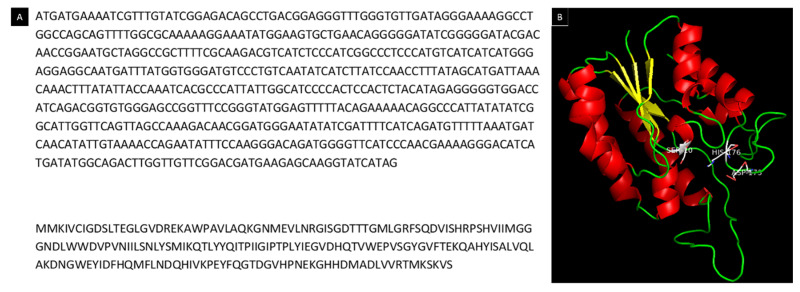
DNA and protein sequences and model of LipB12_A11. (**A**) DNA sequence and translated protein sequence of the DNA conferring lipolytic activity. (**B**) Predicted model of the lipolytic protein LipB12_A11 generated using SWISS-MODEL and visualized with PyMOL using a putative lipolytic protein of the G-D-S-L family from *Desulfitobacterium hafniense* (SMTL ID 4rsh.1.A) as the template. Secondary structures are highlighted in color with α-helices in red, β-sheets in yellow, and ribbons in green. The catalytic triad residues are highlighted (SER10, ASP173, and HIS176).

LipB12_A11 was successfully subcloned using the EcoRV restriction enzyme. The DNA sequence of the subcloned LipB12_A11 lipolytic activity enzyme obtained by primer walking sequencing and the translated protein sequence using ExPASy translate tool are presented in Figure 2A. The predicted protein model using the lipolytic protein G-D-S-L family from *Desulfitobacterium hafniense* DCB-2 (SMTL ID 4rsh.1.A) as the template is provided in Figure 2B. Its Global Model Quality Estimation (GMQE) score was higher than all other templates at 0.62 and its QMEAN quality estimation score was −3.12. Expasy ProtParam predicted a molecular weight of 22241.45 Da and a theorical isoelectric point of 5.64.

### 2.5. Alignment of LipB12_A11 with NCBI Databases for DNA and Protein Sequences

The best match for the DNA sequence in the NR/NT database was the chromosome sequence of *Bacillus citotoxycus* (accession code CP024120.1) with an E-value of 2 × 10^−33^. The query cover was 94% and the percentage of identity was 66.84% with a total score of 156. For the protein database, the best match was a SGNH/GDSL hydrolase family protein from *Bacillus* sp. (accession code WP_028398363.1) with an E-value of 6 × 10^−84^, a total score of 256, query cover of 95%, and identity percentage of 58.73%. BLASTp identified the protein domain architecture as SGNH/GDSL hydrolase family protein: “hydrolytic enzyme such as an esterase or lipase; may have multifunctional properties including broad substrate specificity and regiospecificity”. Indeed, known functions of this protein family are broad and potentially useful in a variety of biotechnological applications [37].

**Figure 3:**
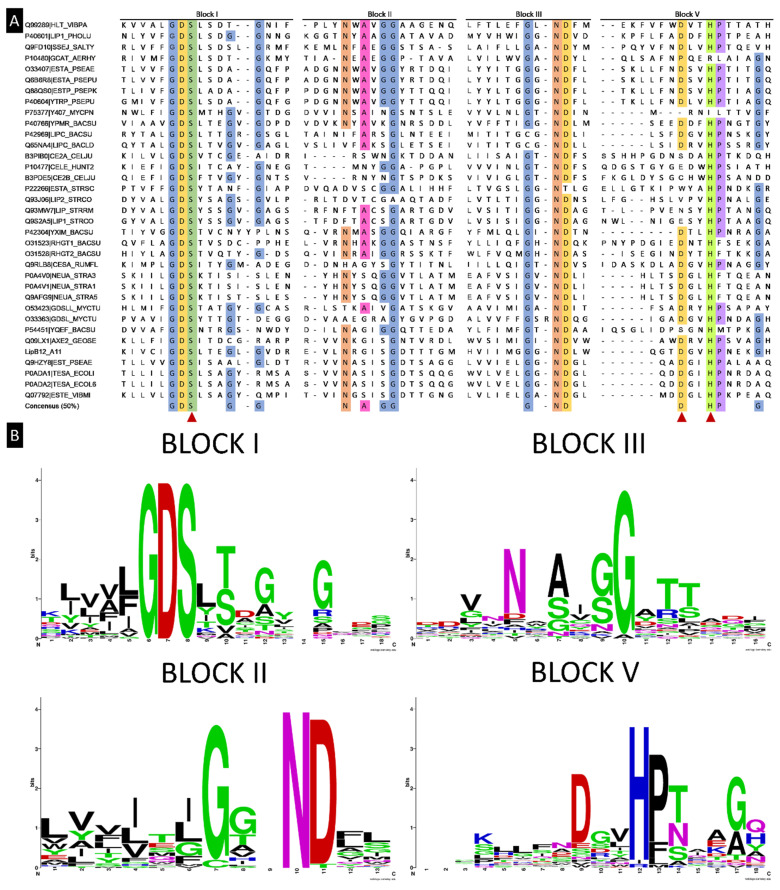
LipB12_A11 sequence analysis. (**A**) Multiple sequence alignment of GDSL family lipases of bacterial origin reviewed and deposited in the UniProt database, including the lipolytic activity enzyme found in this work. Four conserved blocks are represented. A consensus sequence of at least 50% conserved residues among all sequences is given, and the three residues from the catalytic triad are highlighted. (**B**) Protein web logos resulting from the alignments of the amino acids from the four blocks.

A search for reviewed proteins in the UniProt database with the term “GDSL” and with “bacteria” as a taxonomy filter gave 43 results. Manual review of each entry allowed to remove entries that were not true GDSL family proteins (lacking the conserved block of amino acids) and reduced this number to 34 proteins containing the conserved motif. These were submitted for multiple sequence alignment along the lipolytic enzyme found in this study using the Clustal Omega web service. Clustal Omega results of multiple sequence alignments revealed previously described conserved blocks of amino acids in the lipolytic enzyme [37], as shown in Figure 3A. Results were compared to previously described conserved blocks and consensus sequences [37], revealing four conserved blocks previously reported but slight differences in the consensus sequence at a 50% threshold of amino acid occurrences in the same position across all sequences. Moreover, amino acids frequencies in these four blocks were better visualized using the Web Logo tool as shown in Figure 3B.

### 2.6. Purification and Biochemical Characterization of Lipolytic Activity Enzyme LipB12_A11

**Figure 4:**
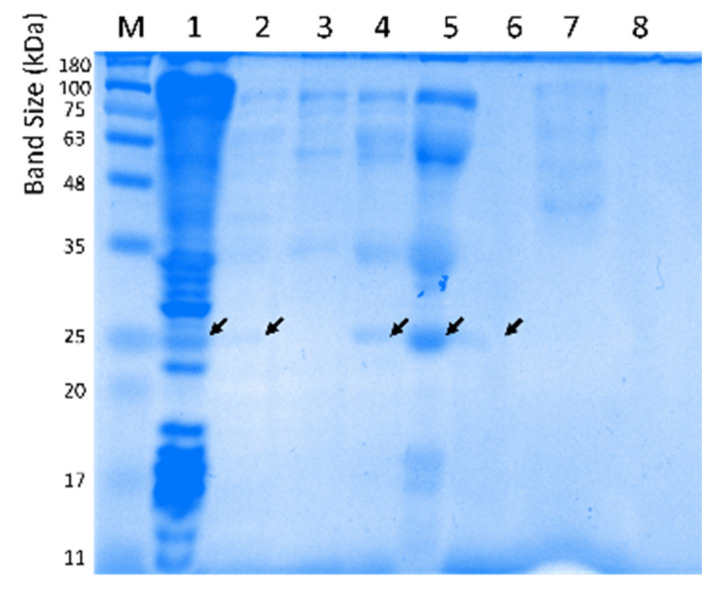
SDS-PAGE of the purification steps for LipB12_A11. (M) NZYColour Protein Marker II (NZYTech, Portugal), (1) crude extract, (2) supernatant after thermal treatment (60 °C, 20 min), (3) pellet from 30% ammonium sulphate precipitation, (4) pellet from 50% ammonium sulphate precipitation, (5) dialyzed and concentrated fraction, (6) pooled molecular exclusion fractions showing lipolytic activity, (7) supernatant from the 50% ammonium sulphate precipitation, (8) concentration column flow-through. Black arrows represent the LipB12_A11 enzyme migration band.

Several different batches of enzyme purification were necessary to perform biochemical characterization. As an example, an overview of the purification steps is given in Figure 4 with an SDS-PAGE of the different fractions and in Table 2 with the protein yields after each step.

#### 2.6.1. LipB12_A11 Temperature and pH Optima and Thermostability

**Figure 5:**
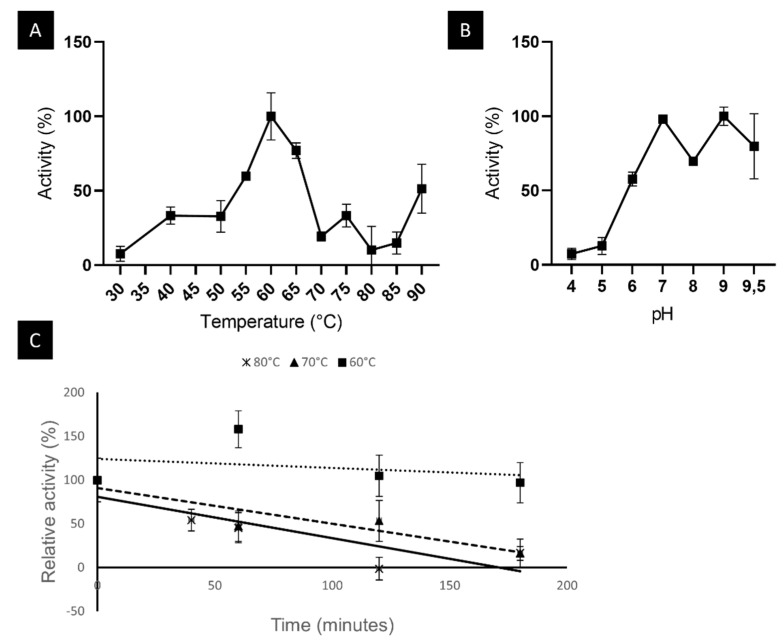
Biochemical characterization of LipB12_A11. (**A**) Optimal temperature for LipB12_A11. 100% activity is 1.58 × 10^−5^ U/µL. (**B**) Optimal pH for LipB12_A11. The 100% activity is 8.70 × 10^−6^ U/µL. (**C**) Thermostability of LipB12_A11. The 100% activity is 9.42 × 10^−6^ U/µL. Solid line: 80 °C linear regression; intermittent line: 70 °C linear regression; dotted line: 60 °C linear regression.

The enzyme was found to have an optimal temperature of 60 °C with the substrate pNP-laurate (C12). The optimal pH was 9 but maintained its activity in the range between 7 and 9.5. The enzyme was found to be thermostable with a half-life of 11.9 h at 60 °C, 1.7 h at 70 °C, and 1.1 h at 80 °C. Results from this biochemical characterization are represented in Figure 5.

#### 2.6.2. LipB12_A11 Substrate Preference

**Figure 6:**
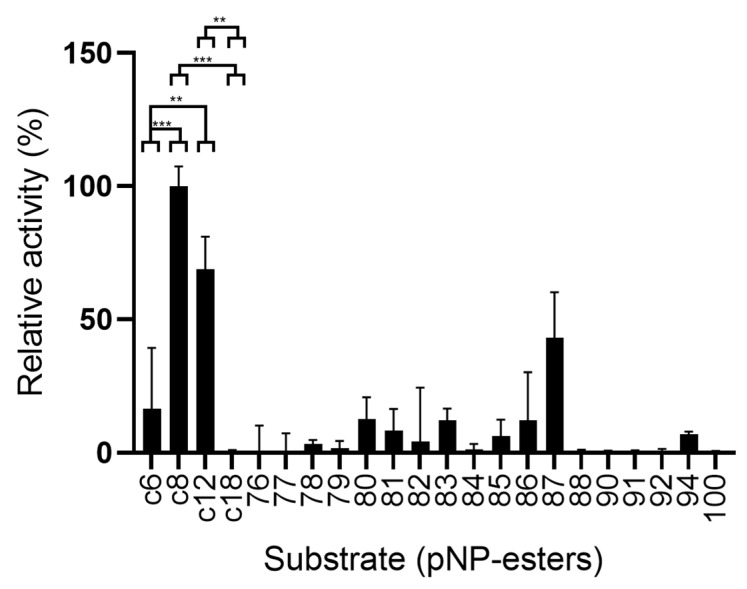
Use of various substrates for LipB12_A11 in relation to its activity towards pNP-octanoate. C6: pNP-hexanoate; C8: pNP-octanoate; C12: pNP-dodecanoate; C18: pNP-stearate; numbers represent synthetic substrates following the naming convention as represented in Supplementary Table S2. The 100% activity is 3.90 ×10^−5^ U/µL. Two asterisks represent a *p*-value < 0.005 and three asterisks represent a *p*-value < 0.0005 for the *t*-test statistical analysis.

Activity was measured using a range of different substrates. The results are summarized in Figure 6. The enzyme was most active using substrates pNP-octanoate (C8), pNP-laurate (C12), and 4-nitrophenyl 5-phenylpentanoate (substrate 87). Some degree of activity was observed towards pNP-hexanoate (C6) and synthetic p-nitrophenyl esters 78, 79, 80, 81, 82, 83, 85, 86, and 94. Longer esters such as pNP-stearate (C18) and the other synthetic compounds assayed were not used as substrates by LipB12_A11.

#### 2.6.3. Effect of Additives and Detergents on LipB12_A11 Enzymatic Activity

**Figure 7:**
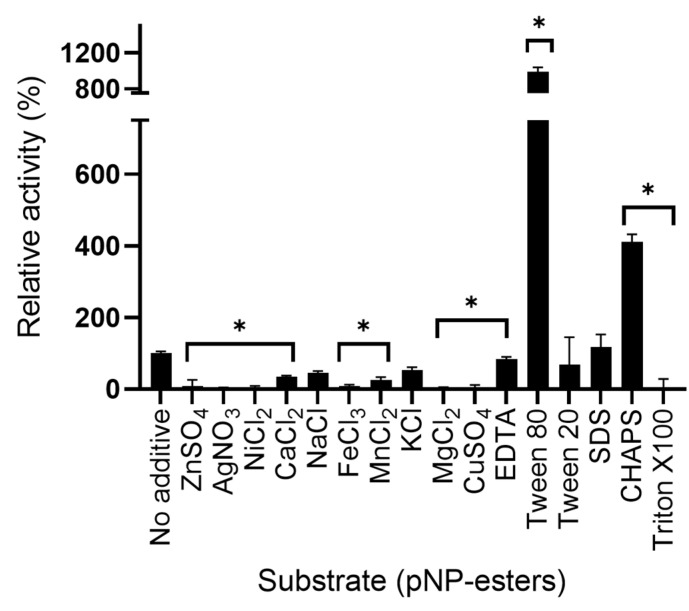
Effect of additives on LipB12_A11 lipolytic activity towards pNP-Laurate relative to the no additive control. Asterisks represent a *p*-value < 0.05 for the t-test statistical analysis. The 100% activity is 1.35 × 10^−5^ U/µL.

The effect of several additives was tested on the LipB12_A11 enzymatic activity. All metal ions decreased the enzymatic activity compared to the no additive control except Na^+^ and K^+^, but activity was also lowered when using the chelating agent EDTA. Ag^+^ and Mg^2+^ completely inhibited the enzymatic activity. Surfactant Tween 80 almost had a 10-fold increase effect on the enzymatic activity. CHAPS also increased the enzymatic activity. On the contrary, Triton X100 inhibited all activity. Tween 20 and SDS had no significant effect on the enzymatic activity. Results on the relative activity of the enzyme with the additives are shown in Figure 7.

#### 2.6.4. LipB12_A11 Enzyme Kinetics

**Figure 8:**
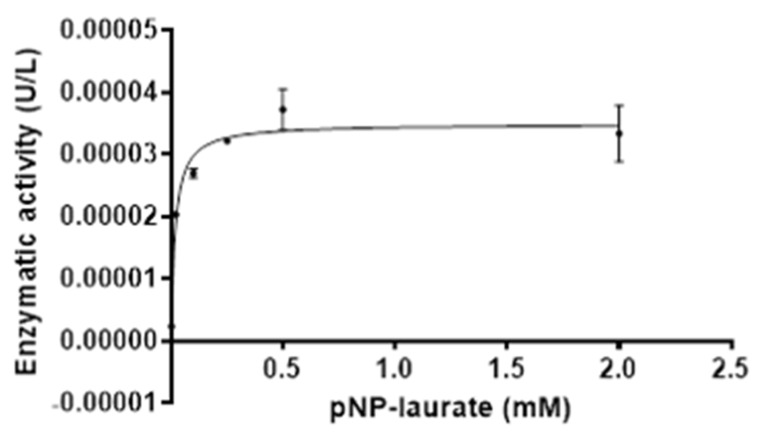
Enzyme kinetics for LipB12_A11 using varying concentrations of the substrate pNP-Laurate at 60 °C and pH 9. Michaelis–Menten non-linear regression was calculated using the GraphPad Prism software.

The enzymatic parameters were calculated for LipB12_A11 using varying concentrations of substrate. Results are represented in Figure 8 showing the Michaelis–Menten fit of the enzyme kinetics and the non-linear regression. The enzyme kinetic parameters with the Michaelis–Menten model were Vmax = 3.487 × 10^−5^ ± 1.091× 10^−6^ U/µL and Km = 0.01731 ± 0.003506 mM, with a coefficient of R^2^ = 0.9425. The Kcat value was 14,362 ± 449 s^−1^.

## 3. Discussion

Functional screenings revealed several positive clones for a diverse range of enzymatic activities that hold biotechnological potential, and that may be thermozymes. Further ongoing studies will allow us to assess these novel biocatalysts and study their biochemical behavior in detail, pointing out the potential for the metagenomic libraries generated in this study to identify undescribed enzymes. One such enzyme is LipB12_A11, that presented lipolytic activity and was successfully subcloned and its ORF identified. As it aligned to a known function protein, its sequence allowed its classification as a SGNH/GDSL hydrolase. Proteins belonging to the SGNH/GDSL hydrolase family [37] contain the conserved GDS(L) amino acids including the active site serine, located near the N-terminus. In the LipB12_A11 sequence, this motif and residue were putatively located in serine 10. Additionally, in this protein family the conserved residues serine, glycine, asparagine, and histidine (SGNH) are present in four conserved blocks numbered I, II, III, and V and play important roles in the hydrolase function [37]. The active site serine is the nucleophile and together with glycine from block II and asparagine from block III (residues Gly42 and Asn71 in LipB12_A11) they act as proton donors for the oxyanion hole. Aspartic acid and histidine from block V are the other two members of the catalytic triad (Asp173 and His176). This histidine residue makes the active site serine more nucleophilic via hydroxyl group deprotonation [37]. One of the key characteristics of this family of hydrolases is the wide range of substrates they can accept. Of the 34 GDSL family proteins of bacterial origin reviewed in UniProt, two belong to thermophilic organisms, namely Cellulase/esterase (CelE) from *Clostridium thermocellum* and Acetylxylan esterase (Axe2) from *Geobacillus stearothermophilus*. In fact, by performing a phylogenetic analysis based on the amino acid sequence of LipB12_A11 to find close proteins, CelE and Axe2 are among the closest proteins, especially Axe2 ( Supplementary Figure S3). Axe2 had a temperature and pH optimum between 50 and 60 °C and 7.1 and 9.2, respectively, using p-nitrophenyl acetate and 2-naphthyl acetate as substrates [38]. The biochemical characterization of CelE is not documented but assays had been performed at temperatures up to 60 °C using CMC and Xylan as substrates at pH 7.0 [39], and at 37 °C using substrates 4-nitrophenyl acetate and acetylated glucomannan at pH 6.5 [40]. Although not included in the reviewed UniProt database, other thermostable GDSL family proteins have been reported, including the GDSL family esterase from *Geobacillus thermodenitrificans* T2 EstL5 with temperature and pH optima at 60 °C and 8.0, respectively, with pNP-butyrate as the substrate [41]; an acetyl xylan esterase from *Caldicellulosiruptor bescii* Cbes-AcXE2 with an optimum temperature of 70 °C and optimum pH of 7.0 using pNP-acetate as the substrate [42]; and a GDSL-type lipase from *Geobacillus thermocatenulatus* Lip29 that had optimal activity at 50 °C and pH 9.5 using pNP-palmitate as the substrate [43]. Our results show that the enzyme LipB12_A11 found in the functional screening of the metagenomic libraries from hot springs possesses comparable temperature optimum to other identified thermostable enzymes belonging to the SGNH/GDSL hydrolase family with an optimum of 60 °C and pH 9 using pNP-laurate as the substrate. Activity in a broad pH range is observed and in agreement with other enzymes of the family [38]. Both substrate preference and presence of an interfacial activation mechanism are instrumental for classification of lipolytic enzymes as either lipases or esterases [43,44,45]. The preference for short substrates such as pNP-octanoate (C8) and pNP-laurate (C12) and lack of activity towards longer chain substrates such as pNP-estearate (C18), coupled with the enzyme kinetics not showing an interfacial activation mechanism typical of true lipases indicates that the SGNH/GDSL hydrolase found in this study is an esterase. The enzyme was found to have some activity towards other alternative synthetic substrates such as 4-nitrophenyl 4-phenylbutanoate, 4-nitrophenyl 2-(pyridin-3-yl)acetate, 4-nitrophenyl linoleate, and especially towards 4-nitrophenyl 5-phenylpentanoate. GDSL family hydrolases are known for their broad substrate specificity and stereoselectivity, associated with a highly flexible active site environment [37]; thus, activity towards many structurally varied nitrophenyl esters was expected, and constitutes a desirable trait for biotechnologically relevant esterases. Regarding its thermostability, the enzyme was highly thermostable at temperature 60 °C with no significant loss of activity after a 3 h incubation period, similarly to other reported thermostable GDSL family esterases [43]. Moreover, a thermal activation effect was observed at that temperature, not unlike what has been observed with other proteins in the family [41]. Activity was heavily affected by the presence of metallic ions, which also has been reported for members of the GDSL family [43], although not to the same extent and with as many metal species as reported here. Lastly, detergents also impacted the activity of the enzyme: CHAPS and most notably Tween 80 enhanced the activity whereas Triton X100 decreased it. As enzymes of this family have numerous potential biotechnological applications, these parameters should be all considered when deciding the best fit for the enzyme applicability.

## 4. Materials and Methods

### 4.1. Sampling

Water was collected from two different hot spring sources in the northwest of Spain, specifically in the city of Ourense, which is located in the Ourense province in the Galicia region. A detailed view of the sampling sites is provided in Supplementary Figure S1, with images generated using the Google Maps service [46].

The hot spring As Burgas (42°20′04.6″ N 7°51′55.1″ W) is located inside the city of Ourense. The site consists of two artificial fountains with a continuous water flow and a hot water pool located above the fountains where public bathing is allowed. Water samples were collected from the continuous flow from one of the fountains (the one located left when facing them) in January 2014.

The hot spring Muiño da Veiga (42°21′05.3″ N 7°54′36.3″ W) is located next to the Miño river in the outskirts of Ourense. The water springs from a manually operated pump, and the site also consists of four hot-water pools open to the public. The water was pumped several times before collection, and the date was December 2015.

Before sample collection, we measured temperature and pH on each site using a thermometer and pH strips. A total of 125 L of water were collected from each source in ethanol-washed plastic bottles to minimize microbial contamination and were washed several times on-site with the hot spring water before final sample collection. The samples were transported to the laboratory on the date of collection for processing in the shortest amount of time possible. In both cases, processing of the samples including the DNA extraction step was completed within the same week as the collection of the samples, starting the next day after collection.

### 4.2. DNA Extraction

The protocol outlined in the commercial kit “Metagenomic DNA isolation kit for water” (Epicentre, Charlotte, NC, USA) was adopted with slight modifications in order to extract the DNA from the microorganisms present in the sample (the metagenomic DNA). This method is based on a combination of chemical and enzymatic treatments for the extraction, allowing the recovery of high-molecular-weight DNA randomly sheared in fragments of approximately 40 kb, which is the optimal size for ligation to the fosmid vector and viral-assisted transformation. We introduced a pre-filtration step using a 5 μm membrane filter in order to remove large particulate material from the As Burgas sample, whereas we found this step not necessary for the Muiño da Veiga sample.

### 4.3. Metagenomic Libraries Construction

A modified procedure for the commercial kit “CopyControl Fosmid Library Production Kit” (Epicentre, USA) was adopted for the construction of both metagenomic libraries.

#### 4.3.1. End Repair and Size Selection of Metagenomic DNA

Ends from the extracted DNA were repaired (blunt-ended and 5′ phosphorylated) following the protocol described by the manufacturer. For size selection, DNA was loaded on a 1% 20 cm agarose gel and electrophoresis was carried out on ice at 40 V overnight with the GeneRuler High Range DNA Ladder (Thermo Fisher Scientific, Waltham, MA, USA) as the molecular weight marker and the Fosmid Control DNA as the reference. External lanes from the gel (including the molecular weight marker, the Fosmid Control DNA, and a small portion of the metagenomic DNA) were cut and stained with a 3x GelGreen (Biotium, Fremont, CA, USA) solution for 30 min in order to visualize the DNA under UV light without exposing the samples to it. The DNA that co-migrated with the 40 kb band was then cut from the gel and the “GeneJET Gel Extraction Kit” (Thermo Fisher Scientific, USA) was used to recover the DNA.

#### 4.3.2. Vector

The fosmid vector pCC1FOS from the kit was replaced with the pCT3FK fosmid (15), a shuttle vector for *E. coli* and *Thermus thermophilus* derived from pCC1FOS, kindly provided by Dr. A. Angelov. A vector map is provided as Supplementary Figure S2 generated with the Serial Cloner software [47]. These vectors contain a single copy replication origin but can be induced to high copy number by arabinose induction mediated by the trfA gene product provided by the host strain and feature a chloramphenicol resistance gene for *E. coli*. The modified vector also adds a thermostable kanamycin resistance gene cassette and sequences from the *T. thermophilus* genome for positive selection and homologous recombination in that host.

#### 4.3.3. Ligation and Packaging of Metagenomic DNA

The ligation of the metagenomic DNA with the empty pCT3FK vector was carried out following the manufacturer’s indications, with an incubation with Fast-Link DNA ligase at 16 °C overnight, and heat inactivation at 70 °C for 10 min. This reaction mixture was used directly to package in Lambda phage extracts. A tittering step was performed testing four serial dilutions (original dilution, 1:10, 1:100, and 1:1000) and it was found that the original dilution produced the desired number of colonies per plate.

*E. coli* strain EPI300^TM^ T1^R^ (F^–^ *mcr*A ∆(*mrr*^-^*hsd*RMS^-^*mcr*BC) ϕ80d*lac*Z∆M15 ∆*lac*X74 *rec*A1 *end*A1 *ara*D139 ∆(*ara*, *leu*)7697 *gal*U *gal*K λ^–^ *rps*L *nup*G *trf*A *ton*A *dhfr*) grown on LB medium consisting of 1% Bacto^TM^ tryptone (BD Biosciences, USA), 0.5% Bacto^TM^ yeast extract (BD Biosciences, San Diego, CA, USA), 0.5% NaCl, and 1.5% Bacto^TM^ Agar (BD Biosciences, San Diego, CA, USA); supplementation with 10 mM MgSO_4_ and 0.2% maltose (to induce the expression of the membrane maltose receptor used by the phage to infect the cell) to an OD_600_ of 0.8 was used in the infection reaction.

For the infection, 10 μL of diluted packaged phage particles were mixed with 100 μL of the culture described above and incubated for 1 h at 37 °C (with no agitation) to allow for the phages to infect the cells. The cells were then spread on LB plates supplemented with chloramphenicol (12.5 μg/mL) and grown overnight at 37 °C. Individual colonies were picked from these plates and transferred to 96-well plates containing 100 μL of LB supplemented with chloramphenicol to constitute the metagenomic libraries.

### 4.4. Functional Screening Methods

#### 4.4.1. Cellulase Screening

The wells from the libraries were replicated on Petri dishes with modified LB media containing 0.5% carboxymethylcellulose (CMC) (endoglucanase) or AVICEL (exoglucanase), chloramphenicol (12.5 µg/mL), and 0.02% (*w*/*v*) arabinose (to induce the vector to high copy number). Activity was detected either with 0.01% trypan blue dye [48] added to the medium, or by staining with 0.1% Congo Red solution for 15 min and destaining with 1 M NaCl solution for another 15 min [49], or alternatively staining with a Gram’s Iodine solution (0.67% KI, 0.33% Iodine) for 5 min [50]. An alternative cellulose substrate was also assayed using 0.1% AZCL-HE-Cellulose (MegaZyme, Wicklow, Ireland) [51] that was added as top agar over LB plates to allow for a better distribution [52]. Yet another substrate was assayed, EnzChek blue fluorescent Cellulase Substrate (Invitrogen, Waltham, MA, USA). A 384-well plate format was adopted, requiring volume adjustments to the suggested protocol from the supplier. The substrate was prepared according to the suggested manufacturer’s protocol, using 50% DMSO to prepare a substrate solution. The digestion buffer used was 100 mM sodium acetate, pH 5. Clones from the libraries were grown at 37 °C for five days to allow natural lysis [53] in 96-well plates containing LB media supplemented with 12.5 µg/mL chloramphenicol and 0.02% arabinose. Cells were centrifuged at maximum speed in a refrigerated (4 °C) centrifuge, and supernatants were employed as crude extracts and transferred to opaque black 384-well plates for fluorescence reading with excitation and emission wavelengths of 360 and 460 nm, respectively. Assay time was 30 min. A threshold for positive hits was adopted requiring readings in the emission wavelength of relative fluorescence units at least above the mean across all wells plus two times the standard deviation [53].

#### 4.4.2. Xylanase Screening

LB media containing 0.1% insoluble Azurine Cross-Linked (AZCL-Xylan) substrate (MegaZyme, Ireland) and supplemented with 0.02% arabinose and 12.5 µg/mL chloramphenicol was used to test the clones for xylanase activity. To maximize the insoluble substrate diffusion in the most homogeneous way possible, this screening media was poured over a previously solidified LB media plate as top agar [54].

#### 4.4.3. β-Xylosidase Screening

LB media containing the specific substrate 0.04% 4-methylumbelliferyl-β-D-xylopyranoside (MUX) and supplemented with 0.02% arabinose and 12.5 µg/mL chloramphenicol were employed for the screening of β-xylosidase activity in the metagenomic libraries [55]. Examination under UV light reveals positive hits due to the release of the fluorophore on the plates.

#### 4.4.4. Lipolytic Activity Screening

For the detection of lipolytic activity, a medium containing 0.5% Bacto^TM^ peptone (BD Biosciences, San Jose, CA, USA), 0.3% Bacto^TM^ yeast extract (BD Biosciences, USA), 1% arabic gum (Acros Organics, Somerville, NJ, USA), 1% (*v*/*v*) glyceryl tributyrate (Sigma-Aldrich, St. Louis, MO, USA), and 1.3% Bacto^TM^ agar (BD Biosciences, USA) was used [56,57,58,59], supplemented with 0.02% arabinose and 12.5 µg/mL chloramphenicol. After addition of glyceryl tributyrate an emulsion was obtained by using a blender for 5 min.

#### 4.4.5. Feruloyl Esterase Screening

A second round of screening for feruloyl esterases was conducted with lipolytic-positive clones from the previous screening as reported in other studies [56,60,61]. Clones were plated on LB medium supplemented with 12.5 µg/mL chloramphenicol and 0.02% arabinose after autoclaving and overlayed with 0.7% agar containing 20 µg/mL of the substrate 4-methylumbelliferyl-trimethylammonium cinnamate chloride (MUTMAC) (Sigma-Aldrich, USA) to further search for feruloyl esterases [60]. Activity was detected under examination with a UV lamp.

#### 4.4.6. β-Glucosidase Screening

For the detection of β-glucosidase activity, liquid LB media containing 1 mM para-nitrophenyl-(β-D)-glucopyranoside (Sigma-Aldrich, USA), 12.5 µg/mL chloramphenicol, and 0.02% arabinose was used. Screening media was dispensed in 96-deep-well plates and each well was inoculated with clones from the libraries. Growth at 37 °C and agitation was sustained for 5 days, cells were precipitated by centrifugation, and 100 µL of supernatant were used to read absorbance at 400 nm wavelength. We adopted the threshold for positives hits as values above the mean absorbance across all wells plus two times the standard deviation [53].

#### 4.4.7. Protease Screening

The EnzChek green fluorescent Bodipy Casein Substrate (Invitrogen, USA) was used to screen for protease activity. We followed the manufacturer’s protocol adapting the format from 96-well plates to 384-well plates (10 µL of working solution consisting of reconstituted substrate in Phosphate Buffer Saline and digestion buffer, and 10 µL of enzyme crude extract). As with cellulase screening using the EnzChek substrate, growth of clones was maintained for 5 days in LB media supplemented with 12.5 µg/mL chloramphenicol and 0.02% arabinose and crude extracts were obtained by centrifugation in a refrigerated centrifuge. Assay time was 1 h, and excitation and emission wavelengths were 485 and 530 nm, respectively. The same threshold of mean across all wells plus two times the standard deviation was adopted for positive hits determination [53].

### 4.5. Subcloning of a DNA Fragment Containing a Lipolytic Activity-Conferring Gene

Digestion with the restriction enzyme *Eco*RV (Roche, Switzerland) was performed on a DNA extraction of a selected clone from the As Burgas metagenomic library (named LipB12_A11) that showed lipolytic activity in the functional screening as described in the previous section. Fosmid DNA was used in a restriction reaction using *Eco*RV with SuRE Cut Buffer B at 37 °C for 1 h. The resulting fragments from the digestion were used for ligation on the pJET1.2 vector following the CloneJET PCR Cloning kit (Thermo Fisher Scientific, USA) instructions. Transformation of the ligation reaction was performed on chemically competent cells, *E. coli* XL1Blue (Agilent, Santa Clara, CA, USA), by the heat-shock method and colonies were cultured on the media outlined for the screening of lipolytic activity, replacing the antibiotic chloramphenicol with ampicillin for positive selection of the new vector and without arabinose. Single colonies showing clear halos on the selection media were selected for further studies.

### 4.6. Sequencing and Primer Walking of a DNA Fragment Containing a Lipolytic Activity-Conferring Gene

Sanger sequencing was performed on DNA extracted with the NZYMiniPrep (NZYTech, Lisboa, Portugal) from lipolytic activity-positive transformants from the subcloning step (sequencing services were provided by Sistemas Genómicos, Valencia, Spain). Primer design for primer walking along the sequence was performed using the NetPrimer analysis software [62] (Premier Biosoft, San Francisco, CA, USA). A list of the primers used is provided in Supplementary Table S1. Primer walking was performed until the forward and reverse sequences overlapped with each other.

### 4.7. Sequence Analysis

The sequence of the ORF was predicted using the ExPASy Translate Tool [63] and aligned to the NR/NT nucleotide collection database from NCBI using the BLAST algorithm [64] on the default settings optimized for “somewhat similar sequences”, and to the NR protein sequences database from NCBI using the BLASTp algorithm on the default settings. The protein sequence structure was modeled with the SWISS-MODEL [65] web tool, using the best matching template provided by the database. The model was then visualized using the PyMol software (Schrödinger LCC, New York, NY, USA) [66]. The protein parameters were estimated using the PROTPARAM tool from ExPASy [67]. Multiple sequence alignments and phylogenetic tree were performed using CLUSTAL OMEGA and Simple Phylogeny, respectively [68], from EMBL-EBI with sequences retrieved from the UniProt Knowledgebase database [69]. Conserved amino acids were visualized using the WEB LOGO service [70] for protein sequences.

### 4.8. Purification

For expression purposes, the LipB12_A12 insert subcloned in the pJET1.2 plasmid was transferred from *E. coli* XL1-Blue (Agilent, USA) to *E. coli* T7 Express (New England Biolabs, USA). DNA was extracted from fresh overnight-grown cultures using the NZYMiniPrep (NZYTech, Portugal) kit and used for transformation using the heat-shock method following protocols outlined previously.

Test were conducted to find optimal induction conditions using varying times, temperatures, and concentrations of IPTG as well as optimal purification step conditions for differential thermal precipitation (times and temperatures of incubation) and ammonium sulphate precipitation (concentration), allowing us to establish an expression and purification protocol outlined below.

A pre-inoculum was prepared by selecting a single colony from an overnight-grown plate of LB media supplemented with ampicillin and transferring to fresh LB medium supplemented with ampicillin. The pre-inoculum was grown overnight and was used to start a culture at initial OD600 of 0.2. This culture was grown to an OD600 of 0.8 and then was induced with 0.04 mM IPTG (final concentration) for 2 h. The cells were pelleted in a refrigerated centrifuge (4 °C) and washed twice using cold milliQ water. The pellet was resuspended in ice-cold sonication buffer 50 mM Tris HCl pH 8.0, EDTA 25 mM, NaCl 25 mM, and sonicated immersed in ice-cold water using a VCX130 Vibra-Cell sonicator (Sonics & Materials INC., Newtown, CT, USA) with a setting of 10 min total time, with pulses of 2 s ON and 8 s OFF to avoid excessive heating and an amplitude of 100%. The cell debris was precipitated using a refrigerated centrifuge (4 °C) and the crude extract was recovered. This crude extract was treated at 60 °C for 20 min for an initial step of purification to precipitate mesophilic proteins from the host and the vector. The thermally treated extract was again centrifuged at 4 °C and the supernatant was recovered. An ammonium sulphate incubation at final concentration 30% (*v*/*v*) for 1 h in a rotatory wheel was performed to remove a fraction of proteins. After a centrifugation at 4 °C, the recovered supernatant was incubated with ammonium sulphate at a final 50% concentration (*v*/*v*) which made the protein of interest to precipitate. Centrifugation at 4 °C allowed to remove the supernatant and recover the pellet that was resuspended in buffer 0.1 M Tris HCl pH 9; 0.1 M NaCl and 1 mM DTT. The same resuspension buffer was used to perform a dialysis with a SnakeSkin Dialysis Tubing membrane with a 3.5 Molecular Weight cut-off (Thermo Fisher Scientific, USA) overnight at 4 °C to remove the ammonium sulphate. Lastly, molecular exclusion chromatography was performed using a HiLoad 16/60 SuperdexTM 75 prep grade column (Cytiva, Marlborough, MA, USA) with a 1 mL/minute flow rate collecting 1 mL fractions. Fractions showing lipolytic activity and without contaminants visualized in SDS-PAGE were combined to perform the biochemical characterization. The pooled fractions were concentrated using a Pierce Concentrator column with 10 K Molecular Weight cut-off (Thermo Fisher Scientific, USA).

### 4.9. Activity Assays

All activity assays were performed in triplicate. Lipolytic activity was tested using 25 mM pNP-Laurate (C12) as the substrate and 0.1 M Sodium Phosphate Buffer pH 7 (at 60 °C) as the reaction buffer. An enzymatic unit is defined as the μmols of p-nitrophenol released per minute and per μL of enzyme in the reaction conditions. A total of 20 µL of purified enzyme were mixed with 730 µL reaction buffer and 100 µL substrate solution and incubated for 30 min at the reaction temperature (60 °C unless indicated otherwise). The reaction was stopped using 250 µL 0.1 M ice-cold NaCO_3_ and transferring to ice. Enzymatic activity was estimated as release of p-nitrophenol which was measured as increments of the absorbance at 400 nm wavelength. The extinction coefficient was previously determined as 17.215 mM/cm [71].

For temperature optima tests, the reaction temperature was changed accordingly for the range between 40 and 90 °C. For pH optima tests, the reaction buffer was 0.1 M Sodium Phosphate Buffer for the range 6–8, 0.1 M Sodium Acetate Buffer for the range 4–6, and 0.1 M Tris HCl Buffer for the range 8–9.5. Thermostability was assessed by incubating the purified enzyme at a specified temperature and for varying amounts of time before performing the activity test at optimal pH and temperature.

A library of synthetic pNP ester substrates [72] was kindly provided by the authors and was also assayed, each substrate at a final concentration of 0.2 mM using the reaction buffer Tris HCl pH 9 (at 60 °C) supplemented with 0.1% Arabic gum and 1% CHAPS. Reaction volumes were adjusted to 5 µL of purified enzyme, 8 µL of substrate, and 387 µL reaction buffer. Supplementary Table S2 shows the chemical formula for these synthetic compounds.

Enzyme kinetics were tested using the following pNP-laurate concentrations: 2, 0.5, 0.25, 0.1, 0.02, and 0.002 mM. Enzyme volume was reduced to 10 µL instead of the 20 µL used in standard assays.

The effect of different additives was tested at a final concentration of 5 mM: NaCl, KCl, CaCl_2_, ZnSO_4_, CuSO_4_, AgNO_3_, FeCl_3_, FeCl_2_, MgCl_2_, MnCl_2_, NiCl_2_, SDS, Triton X-100, Tween 20, Tween 80, CHAPS, CTAB and EDTA.

## 5. Conclusions

This study consisted of the construction and bioprospecting of two metagenomic libraries through functional screening in order to find potentially industrially relevant enzymatic activities. Several positive hits were found for both libraries for some of the activities tested. Further studies were conducted in a selected clone that harbored lipolytic activity, which allowed the classification as a novel esterase in the SGNH/GDSL family of hydrolases, with 60 °C and pH 9 optima using pNP-laurate as the substrate. The enzyme was found to be highly thermostable at 60 °C and its activity can be enhanced with detergents such as Tween 80. As enzymes of this family have numerous potential biotechnological applications, these parameters should all be considered when deciding the best fit for the enzyme applicability.

## Figures and Tables

**Table 1:** Overview of all the functional screenings performed on the two metagenomic libraries and the number of positive hits detected for each specific substrate.

Enzymatic Activity	Substrate	Method	As Burgas	Muiño da Veiga
Endoglucanase	CMC	Halo	0	0
	AZCL-HE-Celulose	Colorimetric	0	0
	EnzChek cellulase	Fluorimetric	2	4
Exoglucanase	AVICEL	Halo	0	0
β-glucosidase	pNP-glucopyranoside	Colorimetric	5	1
Xylanase	AZCL-Xylan	Colorimetric	0	0
β-xylosidase	MUX	Fluorimetric	2	0
Protease	EnzChek Casein	Fluorimetric	4	6
Lipolytic	Tributyrin	Halo	1	8
Feruloyl esterase	MUTMAC	Fluorimetric	0	0

**Table 2:** Purification steps for LipB12_A11.

Purification Step	Volume (mL)	Total Activity (U/µL)	Total Protein (mg)	Specific Activity (U/mg)	Yield (%)	Purification Fold
Crude extract	16	0.000201	17.01	1.1807 × 10^−8^	100	1
Differential thermal precipitation	8	0.000155	10.88	1.4287 × 10^−8^	77.42	1.21
Ammonium sulphate precipitation	8	0.000149	4.33	3.4641 × 10^−8^	74.69	2.93
Molecular exclusion chromatography	3	0.000145	2.70	5.383 × 10^−8^	72.49	4.56

## Data Availability

Not applicable.

## Supplementary Materials

The following supporting information can be downloaded at: https://www.mdpi.com/article/10.3390/ijms23105733/s1.
