# Supplementary material for: Bioprospecting for Thermozymes and Characterization of a Novel Lipolytic Thermozyme Belonging to the SGNH/GDSL Family of Hydrolases
Source: Int J Mol Sci. 2022 May 20;23(10):5733. doi: 10.3390/ijms23105733 (PMC9145741; doi:10.3390/ijms23105733)
Supplement: Supplementary file 1 [file ijms-23-05733-s001.zip › ijms-1685496-supplementary.pdf]

Supplementary Materials:

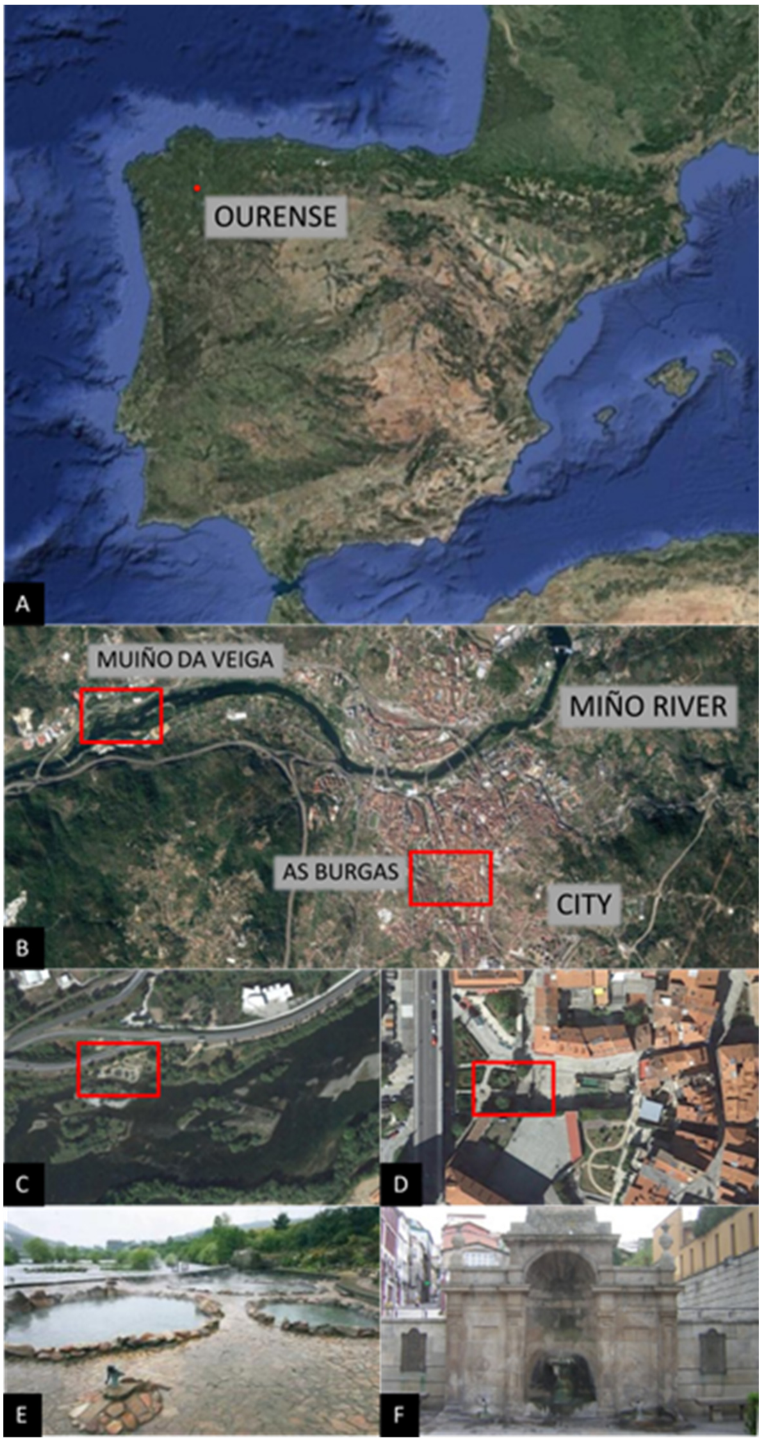

Figure S1. Sampling sites locations. (**a**) Location of the city of Ourense in the Iberian Peninsula, marked as a red dot. (**b**) Close-up view of the city of Ourense and the Miño river north of it. Inside red squares are the sampling sites, Muiño da Veiga to the left and As Burgas to the right of the image. (**c**) Close-up view of the red square from the previous picture corresponding to the Muiño da Veiga hot spring. Another red square marks the precise location of the hot spring. (**d**) Close-up view of the red square from Figure 1B that corresponds to the As Burgas hot spring. Another red square marks the exact location of the As Burgas hot spring fountains. (**e**) Muiño da Veiga hot spring, on the center of the image the four main pools can be viewed, in the lower part of the image is the fountain where water was collected, in the upper left corner the Miño river can be seen. (**f**) As Burgas hot spring, in the lower part of the image two fountains are present. Water was collected from the left fountain.

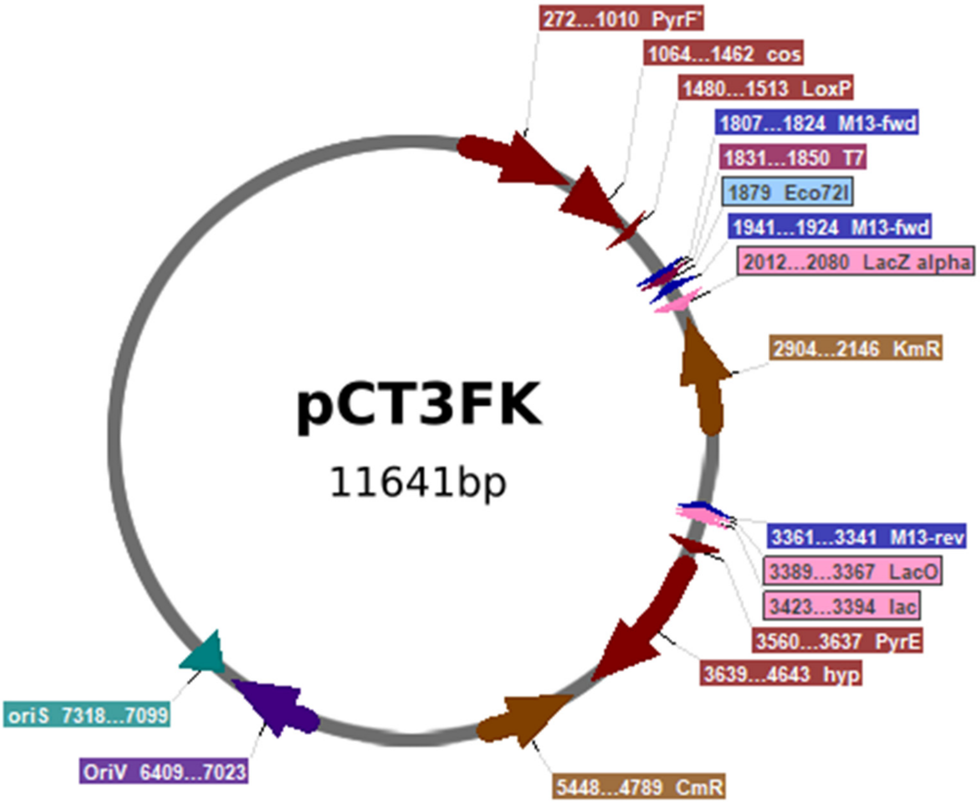

Figure S2. The pCT3FK fosmid vector map, highlighting some of its features, including PyrF' and PyrE for *T. thermophilus* recombination, Km^R for kanamycin resistance in that host, restriction site *Eco*72I, Cm^R for chloramphenicol resistance in *E. coli*, origins of replication OriS and OriV for the bacterial F plasmid and cos site for lambda phage recognition.

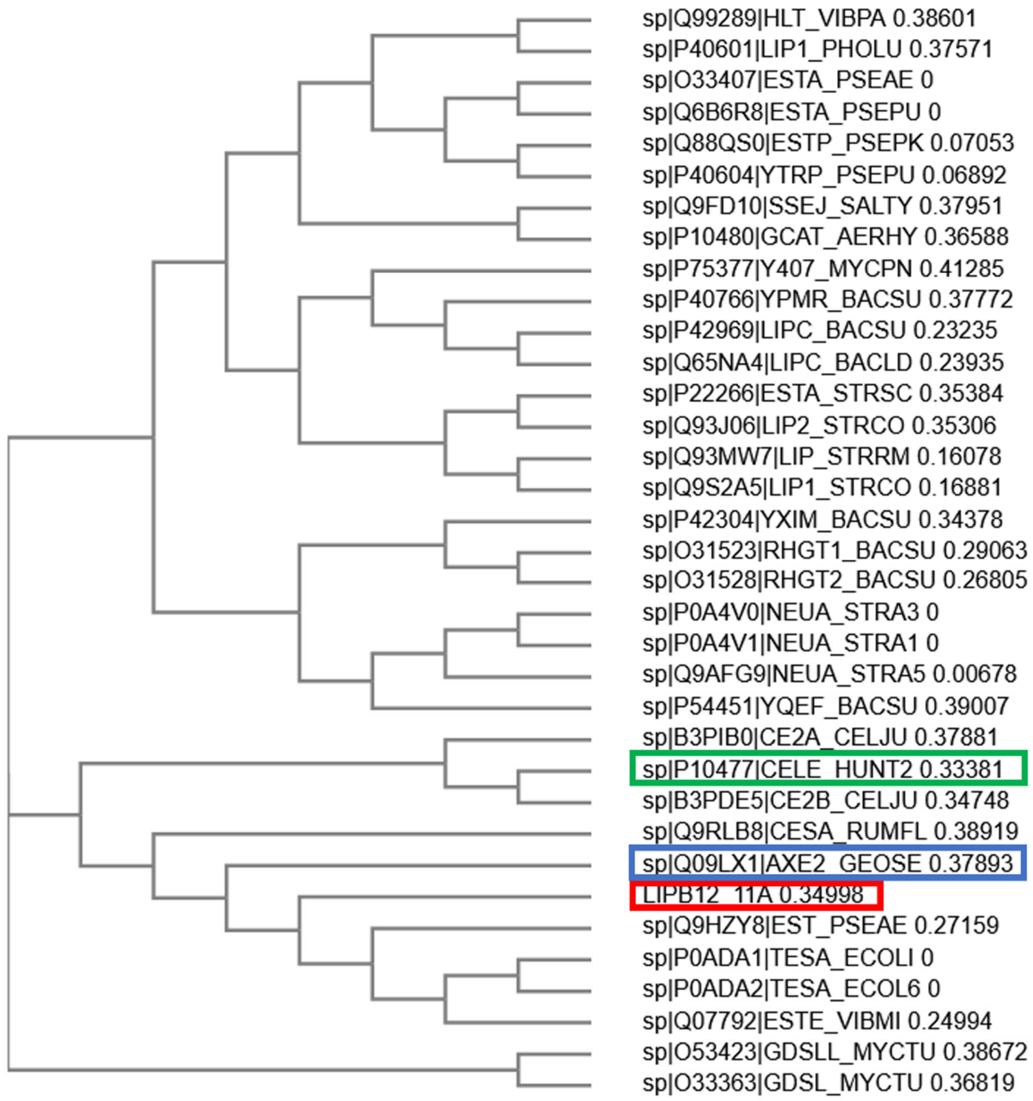

Figure S3. Phylogenetic tree of the LipB12_A11 (indicated with a red rectangle) performed with Simple Phylogeny from EMBL-EBI and using the Neighbour-joining method as clustering method. Cellulase/esterase (CelE) from *Clostridium thermocellum* (green rectangle) and Acetylxylan esterase (Axe2) from *Geobacillus stearothermophilus* (blue rectangle) are indicated.

Supplementary Table S1. Sequencing primers used in the primer walking sequencing process for the lipolytic activity conferring DNA cloned fragment.

**Primer**	**Sequence (5′ – 3′)**
pJET1.2 Forward	CGACTCACTATAGGGAGAGCGGC
pJET1.2 Reverse	AAGAACATCGATTTTCCATGGCAG
PF1	GGATTTACTGTATGACGGGATACCT
PR1	AGTCGCACTACACGGTCGTTCTGTT
PF2	GGAGCGGCTTTATTTTACCCACTTT
PR2	CAGAAACAAGGTTGGGACAAGCACT
PF3	CATCCGTTGTCTTTGGCTAACTGAA
PR3	TCTCAACATAAGGAAAGCGAAACG
PF4	ATGACTGGAAAAGCAAGGGGAATCT
PR4	GATACCGAAACGATGACAGCCCTTA
PF5	ATTAGGGGATTCATCAGGGCACAGT
PR5	ATCGCTTGTAAGACGGAGGGAATA

Supplementary Table S2. Synthetic substrates used in this study.

Code	Substrate	Chemical formula
76	4-nitrophenyl 2-(4-isobutylphenyl)propanoate)	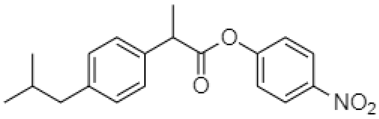
77	4-nitrophenyl 3-phenylpropanoate	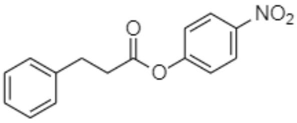
78	4-nitrophenyl cinnamate	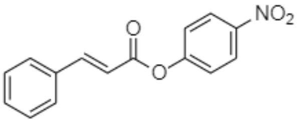
79	4-nitrophenyl benzoate	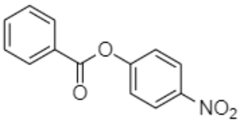
80	4-nitrophenyl 4-(1H-indol-2-yl)butanoate	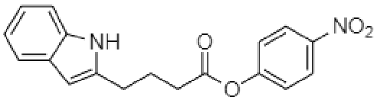
81	4-nitrophenyl 4-phenylbutanoate	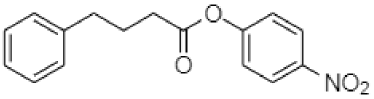
82	4-nitrophenyl 2-(naphthalen-1-yl)acetate	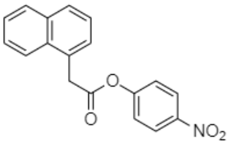
83	4-nitrophenyl 2-(pyridin-3-yl)acetate	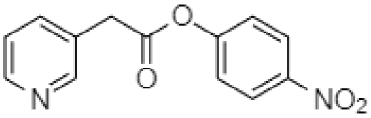
84	4-nitrophenyl oleate	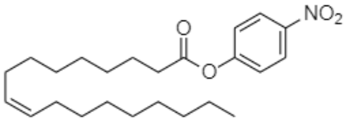
85	4-nitrophenyl 3-(pyridin-3-yl)propanoate	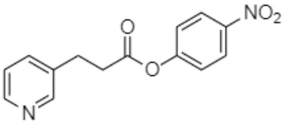
86	4-nitrophenyl 2-(6-methoxynaphthalen-2-yl)propanoate	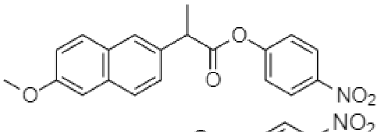
87	4-nitrophenyl 5-phenylpentanoate	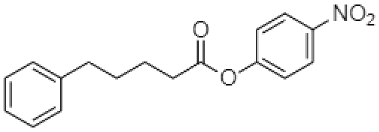
88	4-nitrophenyl cyclohexanecarboxylate	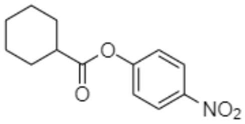
90	4-nitrophenyl 3-methylbutanoate	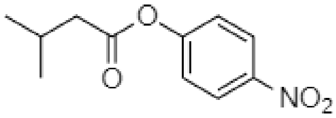

91	4-nitrophenyl 2-methylheptanoate	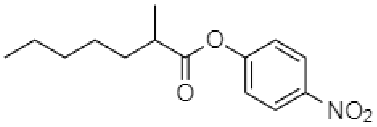
92	4-nitrophenyl (E)-3-(3,4-dimethoxyphenyl)acrylate	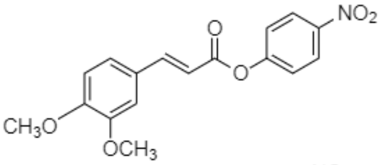
94	4-nitrophenyl linoleate	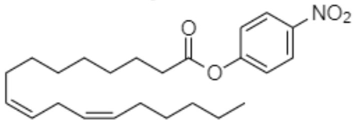
100	(1R,3R)-4-nitrophenyl 3-(2,2-dibromovinyl)-2,2-dimethylcyclopropanecarboxylate	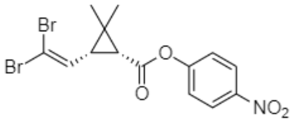
